# Assessment of Gate Width Size on Lifetime-Based Förster Resonance Energy Transfer Parameter Estimation

**DOI:** 10.3390/photonics2041027

**Published:** 2015-09-28

**Authors:** Sez-Jade Chen, Nattawut Sinsuebphon, Xavier Intes

**Affiliations:** Department of Biomedical Engineering, Rensselaer Polytechnic Institute, 110 8th Street, Troy, NY 12180, USA

**Keywords:** Förster Resonance Energy Transfer (FRET), time-resolved imaging, gate width, fluorescence lifetime, gated ICCD, near infrared (NIR) dyes, *in vivo* imaging

## Abstract

Förster Resonance Energy Transfer (FRET) enables the observation of interactions at the nanoscale level through the use of fluorescence optical imaging techniques. In FRET, fluorescence lifetime imaging can be used to quantify the fluorescence lifetime changes of the donor molecule, which are associated with proximity between acceptor and donor molecules. Among the FRET parameters derived from fluorescence lifetime imaging, the percentage of donor that interacts with the acceptor (in proximity) can be estimated via model-based fitting. However, estimation of the lifetime parameters can be affected by the acquisition parameters such as the temporal characteristics of the imaging system. Herein, we investigate the effect of various gate widths on the accuracy of estimation of FRET parameters with focus on the near-infrared spectral window. Experiments were performed *in silico*, *in vitro*, and *in vivo* with gate width sizes ranging from 300 ps to 1000 ps in intervals of 100 ps. For all cases, the FRET parameters were retrieved accurately and the imaging acquisition time was decreased three-fold. These results indicate that increasing the gate width up to 1000 ps still allows for accurate quantification of FRET interactions even in the case of short lifetimes such as those encountered with near-infrared FRET pairs.

## 1. Introduction

Förster Resonance Energy Transfer (FRET) is a phenomenon involving the non-radiative transfer of energy between an excited molecule of higher energy (donor) and one of lower energy (acceptor) [1,2]. This interaction only occurs when the molecules are approximately 2–10 nm apart, a distance that is comparable to the scale of biological interactions at the molecular level [3], and when there is overlap between the spectra of the two molecules. On transmission of energy to the acceptor, the fluorescence lifetime of the donor is reduced and its fluorescence emission intensity decreases. It is possible to use both intensity and lifetime imaging to establish the occurrence of FRET, but lifetime imaging benefits from instrumental implementation of single wavelength excitation/detection, independence from local intensity or concentration, and limited effect of background optical properties for *in vivo* imaging [4]. Lifetime imaging allows us to quantitatively retrieve the donor molecule populations that are free and those that are interacting with the acceptor within the sample [5,6]. The use of FRET for *in vitro* studies is already well established [7,8], and researchers have begun to establish the proper techniques for *in vivo* studies [9–12].

However, the ability to visualize fluorescence within an *in vivo* sample is limited by the absorption and scattering of the incoming light within the tissue. For intact animal tissues, the absorbance of biological substances such as water and hemoglobin is highest for wavelengths between 200 nm and 650 nm [13,14], which are within the visible region. Researchers have been using visible fluorescence as a marker for many years with some variants of GFP [15], such as cyan and yellow FPs (CFP, YFP respectively) employed for FRET experiments *in vitro* [16]. These fluorophores are excited and emit energy in the visible range, which severely limits the depth of interrogation, and also leads to low image resolution and high background fluorescence due to scattering [17]. In order to enable visualization of deep tissues, we instead perform imaging in the near infrared (NIR) region between 600 nm and 1000 nm [14,18,19]. The reduced scattering and absorption properties of biological tissues in this spectral window allow for deeper penetration of light into thick tissues, such as the bodies of small animals, without need for invasive methods such as dissection, biopsy, or complex and expensive models such as intravital chambers [19,20]. However, most of the NIR fluorophores produced to date have lower efficiency and shorter lifetimes (typically less than 1.5 ns) than visible fluorophores (a few nanoseconds), and thus could be more difficult to image with established techniques such as those currently employed in microscopy [5,21].

Fluorescence lifetime imaging microscopy (FLIM) data can be acquired in either the frequency domain (FD) or the time domain (TD). In FD-FLIM, a sinusoidally modulated source is used, and the phase shift between the excitation light and the emitted fluorescence is used to determine the lifetime. For wide-field imaging in low-light settings, TD-FLIM is preferred over FD-FLIM techniques. Hence, FD-FLIM is not used in this work and the reader is encouraged to refer to [22] for more information. In TD-FLIM, a pulsed light source is used and fast detectors record the build-up of the statistical temporal profile of fluorescence emission (time point spread function—TPSF). For fast time-resolved detection, one can use either time correlated single-photon counting (TCSPC) or a gated-integration approach. TCSPC is efficient and provides high signal-to-noise ratio (SNR), but has longer acquisition time and is typically associated with a single detector acquisition scheme [22–24]. Conversely, time-gated systems allow for dense spatial acquisition but are relatively inefficient in photon collection. Time-gated systems are built around a CCD camera coupled with a fast optical gating system that can be opened at specified gate delays and for specific lengths of time. When the CCD is triggered by a laser pulse, the CCD is “gated-on” and photons are collected at the CCD. The amount of time that the CCD is in this “gated-on” state is referred to as the gate width. Scanning of the gate delays allows for acquisition of the entire TPSF at each pixel of the camera.

Selecting a proper gate width is of importance for all biomedical optics applications. For instance, Sharman *et al*. [25] demonstrated that larger gate width led to increased photon counts and improved accuracy of lifetime estimation in mono-exponential cases. Conversely, Venugopal previously demonstrated that increasing the size of the gate results in a broadening of the instrument response function (IRF) characteristic of the system and increased difficulty in estimation of optical properties (μa, μs’) of a sample [26]. For model based fitting, Ma *et al.* [27] demonstrated that if the lifetime is ~1.5 times larger than the IRF, accurate lifetime fitting can be obtained without convolution of the IRF. However, in the case of FRET, a double exponential behavior is expected with a short lifetime component. Zhong *et al.* [28] showed that picosecond-gating is a useful approach for imaging FRET visible fluorophores with lifetimes in the nanosecond-range. To the best of our knowledge, the effect of gate width on estimation of the FRET lifetime parameters in the NIR region has not yet been reported. Herein, a systematic investigation of the impact of gate width size upon the accuracy of the lifetime model-based FRET quantification is investigated within fluorophore parameters relevant to the NIR spectral range. Simulations were performed over a wide range of reduced lifetimes associated with the FRETing donor population, as well as different FRETing donor population percentages. These synthetic data were computed for gate widths ranging from 300 ps to 1000 ps, which are the typical values encountered in the literature and associated with commercially available gated ICCD systems. Model-based fitting was performed to estimate the FRETing parameters and to assess the impact of gate-width on fitting accuracy. *In vitro* and *in vivo* experimental validation was performed using an NIR donor and acceptor FRET pair at different ratios.

## 2. Materials and Methods

### 2.1. Time-Resolved Optical Tomography System

**Figure 1:**
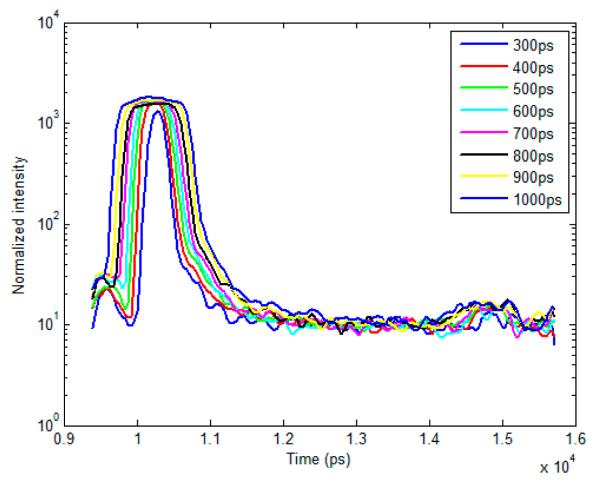
Experimental IRFs acquired from our wide-field imager at each gate width tested in this study.

The setup of the time-resolved optical tomography system used in this study was described in detail by Venugopal *et al.* [26,29]. Briefly, a Ti-Sapphire MaiTai HP laser (Newport Spectra-Physics, Irvine, CA, USA) is used to provide 100 fs pulses at a repetition rate of 80 MHz, and a digital micromirror device (DMD) is used to modulate the light entering the sample. A portion of the light is then used to trigger a gated intensified charge coupled device (ICCD) camera (PicoStar HR, LaVision GmbH, Goettingen, Germany) for collection of fluorescence images in both reflectance and transmittance geometries. In this study, the gate width of the ICCD is increased from 300 ps to 1000 ps in 100 ps intervals through control of the ICCD using the DaVis software (LaVision GmbH, Goettingen, Germany). The IRF was acquired by putting a slightly diffusing white paper in the imaging plane. The temporal profiles of each gate width, normalized to have the same maximum value, are shown in Figure 1 (log-scale). For all experiments and for collection of the IRFs, the step size between gates was set at 40 ps and the laser excitation wavelength was set to 695 nm. The exposure time and laser power were changed to produce optimal signal on the sample, and datasets were taken at each gate width in order to determine the effect on FRET fluorescence decay. *In silico* parameters were selected to match this experimental setup. For *in vitro* and *in vivo* studies, a 715 nm long-pass filter (FF01-715/LP-25, Semrock, NY, USA) and a 720/13 nm bandpass filter (FF01-720/13-25, Semrock, NY, USA) were used to filter the light detected at the ICCD during collection of the TSPFs.

### 2.2. Bi-Exponential Model

Since two populations of donor molecules, those bound to acceptor and those unbound in solution, are present during a particular FRET interaction [5], the fluorescence decay follows a bi-exponential model:

(1) $$I(t)=\left[\mathrm{IRF}(t)⊗(A_{1}e^{-\left(\frac{t}{\tau 1}\right)}+A_{2}e^{-\left(\frac{t}{\tau 2}\right)})\right]+\mathrm{Noise}$$

where *I*(*t*) is the fluorescence TPSF, *IRF*(*t*) is the instrument response function, *t* is the time, and ⊗ represents convolution. *τ*_1_ and *τ*_2_ are the lifetime coefficients while *A*_1_ and *A*_2_ are the amplitude coefficients of the FRETing and non-FRETing donor molecules respectively. Poisson noise is added after the convolution to simulate the noise incurred during an experiment due to photon (“shot”) noise [30,31]. Since *A*_1_ and *τ*_1_ represent the percentage of donor that participates in FRET and its fluorescence lifetime, we focus on reporting the estimation of these two parameters in this work.

### 2.3. In Silico Analysis of Varying A:D Ratios and Lifetimes

TPSFs were formed using Equation (1) to simulate different Acceptor-to-Donor (A:D) ratios and fluorescence lifetimes by varying the FRET donor fraction *A*_1_ and the lifetime *τ*_1_. The *A*_1_ value was varied from 10% to 90% in intervals of 10%, which represents an increase in the percentage of donor that interacts with acceptor in the sample. The *τ*_1_ value was varied from 200 ps to 400 ps in intervals of 50 ps to simulate various fluorescence lifetimes that are common for NIR fluorophores in the quenched state. The lifetime of the unquenched donor was set to *τ*_2_ = 1 ns over the whole study. This lifetime matches that of AlexaFluor 700 (LifeTechnologies, NY, USA) *in vivo*, the donor used in the experimental validation. These decay curves were then convolved with the IRFs collected experimentally at the given set of gate widths, after which random Poisson noise was added.

During the creation of the models, two experimental conditions were simulated: “optimal” and “low-light.” Under optimal experimental conditions, the exposure time and laser power within the imaging system are adjusted to provide the maximal amount of photons in the region of interest without saturation of the CCD. The maximum photon count in this case is approximately 3000 photons for the system mentioned in this work (12-bits). In this condition, the SNR is optimal for all data acquired over the field-of-view. This approach is experimentally achieved by using active wide-field illumination as described in References [10,32,33]. In low-light conditions, the signal was normalized to a maximum of 1000 photons at the CCD. Two hundred TPSFs were simulated for each gate width with the maximum intensity of the TPSF at either 3000 photons or 1000 photons. This threshold was set as double-exponential fitting is notoriously difficult below such photon counts [34].

To retrieve the lifetime and fractional abundance of the quenched donor population, the asymptotic portions of the TPSF were fit with the bi-exponential model. Techniques such as rapid lifetime determination (RLD) are commonly employed with visible fluorophores, but they are not accurate when the IRF temporal width is not negligible compared to the considered lifetime [35]. In the NIR, the IRF temporal width is on the same order as the quenched donor lifetime or even larger. Hence, we employ a classical model-based fit of the TPSF using the linear least-squares (LSQR) method [30].

### 2.4. In Vitro Experiments Using an NIR FRET Pair Conjugated with Mouse Antibodies

AlexaFluor 700 and AlexaFluor 750, conjugated to mouse immunoglobin G (IgG) were purchased directly from LifeTechnologies. Solutions with various mixture ratios of FRET donor-acceptor pairs were prepared in a 96-well plate and imaged at the specified gate widths. These two dyes were chosen because they provided the best lifetime-based FRET measurements in the NIR range among various other potential FRET pairs [36]. Both labels were first diluted to a concentration of 50 μg/mL using PBS buffer and then pipetted into the wells in A:D ratios of 0:1, 1:1, 2:1, and 3:1. As shown previously, intermolecular FRET (or FRET fraction of FRETing donor) is linearly dependent on the A:D ratios [9,37]. After 30 minutes, images were taken at each gate width while varying the exposure time and laser power to produce optimal signal. For data processing, a region of interest (ROI) defined as a 14 × 14 pixel area was employed for each well, leading to 196 pixels (TPSFs) for every condition tested.

### 2.5. In Vivo Experiments with Murine Models

All *in vivo* procedures were performed according to the protocols of the Institutional Animal Care and Use Committee (IACUC) at Rensselaer Polytechnic Institute. T47D breast cancer cells were injected subcutaneously into the mammary fat pads of a mouse to create a tumor model that developed for a few weeks, as in the protocol described by Abe and colleagues [12]. After substantial tumor growth (~3 × 3 × 3 mm^3^), the animal was injected through the tail-vein with a mixture of transferrin (Tfn) probes labeled with AF700 or AF750 in an A:D ratio of 2:1. Transferrin was used because it is a common carrier for anti-cancer drugs in the clinical field [38] and it is native to all tissues in the body. Six hours after injection, the mouse was anesthetized and imaged at different system gate widths at an excitation wavelength of 695 nm to visualize FRET interactions. Following data collection, the organs were isolated in a region of interest and analyzed with bi-exponential fitting algorithms written in MATLAB (Mathworks, Natick, MA, USA). This algorithm uses least-squares methods to find a bi-exponential fit for a given decay curve and returns the FRET lifetime parameters. For this work, we focused on two organs: the liver and the tumor. As described in Reference [10], both from known biology and *ex vivo*/*in vivo* data validation, the liver exhibits a high amount of FRET since it has a rich level of expression in transferrin receptors. The tumor represents a case of targeted delivery due to increased angiogenesis and a higher number of receptors for transferrin at the tumor site than in normal tissue. Increased FRET signal within the tumor area would be indicative of uptake of transferrin through receptor-mediated endocytosis [38,39].

### 2.6. Fitting Methods Using Bi-Exponential Model

Throughout this study, a least-squares method was used to fit the TPSFs to the model in Equation (1) in order to retrieve the FRET donor fraction *A*_1_ and the quenched lifetime *τ*_1_. The long lifetime *τ*_2_ was fixed to 1.0 ns, the unquenched lifetime of AlexaFluor 700 as provided by the manufacturer. Since the quenched lifetime *τ*_1_ is unknown, an initial guess of 280 ps was used and the parameter was fit within ±50 ps. These values were chosen based on our previous experience [12]. The fit was performed on the asymptotic part of the TPSF, from 95% of the peak to 1% of the peak (typically ~130 gates) for robustness [30]. The reported *A*_1_ and *τ*_1_ values are the average of the individual fit results for each pixel within the full region of interest. During the process of lifetime estimation *in vitro* and *in vivo*, only the TPSFs with maxima above 1000 photon counts were analyzed to stay consistent with the *in silico* study. All fitting parameters were kept constant over the whole study (*in silico, in vitro* and *in vivo*).

## 3. Results and Discussion

In this study, the effect of gate width was tested *in silico* through creation of simulated TPSFs, *in vitro* in a well-plate using IgG labeled with FRET donors and acceptors, and *in vivo* in a murine tumor model to provide a comprehensive analysis of the quantification of FRET under various experimental conditions.

### 3.1. In Silico Investigation

**Figure 2:**
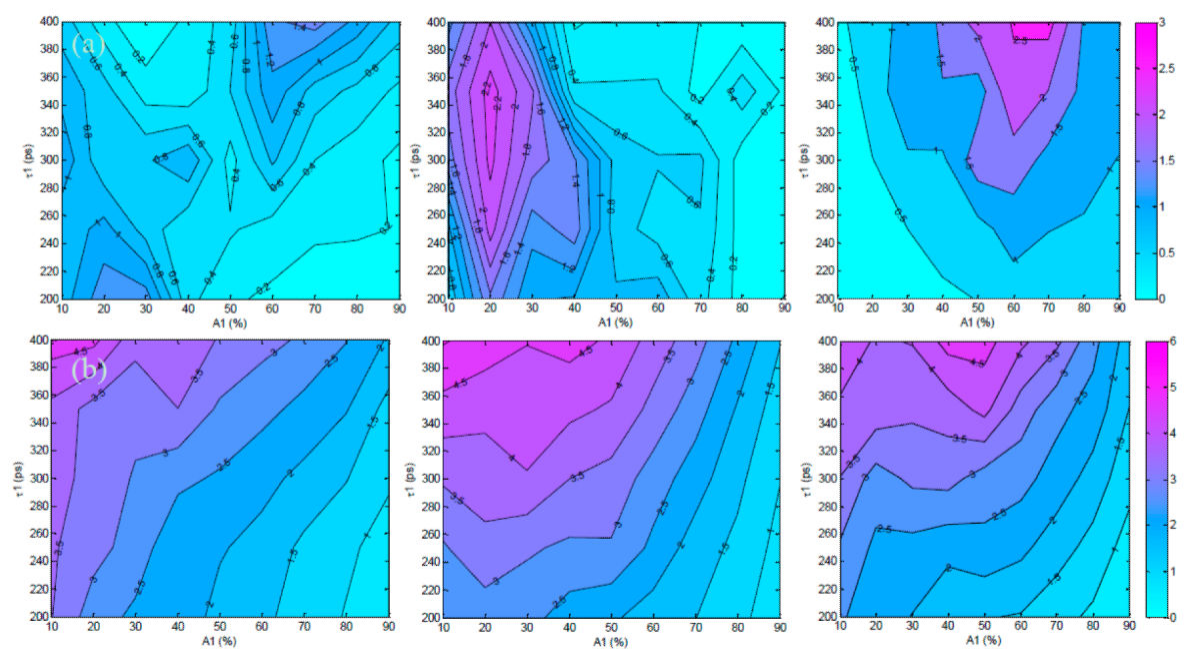
Trends in (**a**) absolute error and (**b**) standard deviation for estimation of *A*_1_ under optimal conditions (maximum of the TPSF at 3000 photons) and 300 ps, 600 ps, 1000 ps gates.

As described previously in Section 2.3, the first set of simulated TPSFs was performed with optimal conditions in which the maximum photon count was 3000 photons. Figure 2a,b show the absolute error and standard deviations for estimations of *A*_1_ (varied from 10% to 90%) and *τ*_1_ (varied from 200 ps to 400 ps) for the 300 ps, 600 ps, and 1000 ps gate widths. The absolute estimation errors were less than 3% for all tested parameters, with the highest error for the 1000 ps gate. As the gate width increased, the maximum error increased as well (1.4% at 300 ps, 2.2% at 600 ps and 2.6% at 1000 ps). However, the distributions of error were different for each gate width. For the 600 ps gate the error was highest at *A*_1_ ≤ 30%, while the error was highest at *A*_1_ ≥ 60% for the 1000 ps gate. For the 300 ps gate the error was highest at both of these regions.

Overall, the standard deviations were less than 10% for all cases, with the highest deviations at the lowest *A*_1_ values for all tested lifetimes. A low value of *A*_1_ (*A*_1_ ≤ 10%) means that there is less donor participating in FRET within the sample, which would be difficult to quantify no matter the lifetime of the fluorophore. The standard deviations are seen to slightly increase with gate width for the three cases shown in Figure 2. Notably, *τ*_1_ has less effect on parameter accuracy than *A*_1_ because the quenched lifetime should be independent of concentration and intensity [22,31].

**Figure 3:**
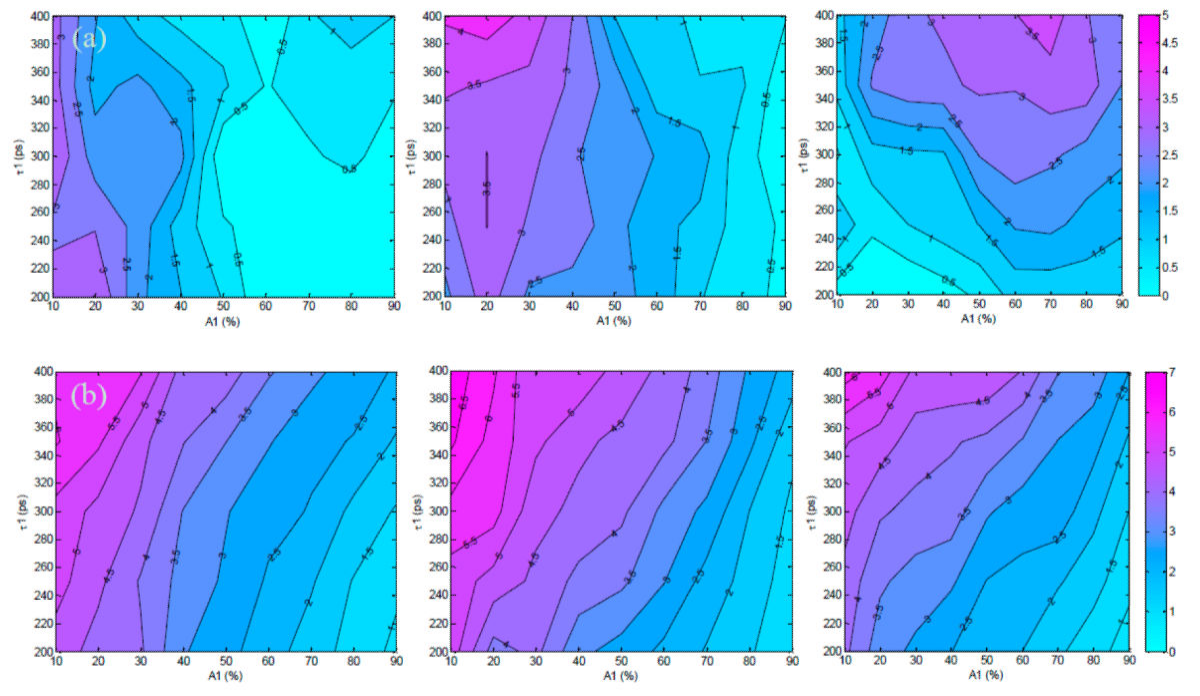
Trends in (**a**) absolute error and (**b**) standard deviation for estimation of *A*_1_ under low-light conditions (maximum of the TPSF at 1000 photons) and 300 ps, 600 ps, 1000 ps gates.

The second set of synthetic TPSFs was set with low-light, or photon-starved, conditions in which the maximum count was 1000 photons. Figure 3a,b summarizes the absolute error and standard deviations of estimation of *A*_1_ for the low-light conditions and a 300 ps, 600 ps, and 1000 ps gate. The overall results in the low light conditions follow the same trend as in the optimal conditions, with an increase in observed errors and standard deviation as expected. The maximum absolute errors for this case were ~4.3%. A similar trend of increased deviation at low values of *A*_1_ is also observed, with a maximum deviation of 6.8% and increasing deviation for higher gate widths.

**Figure 4:**
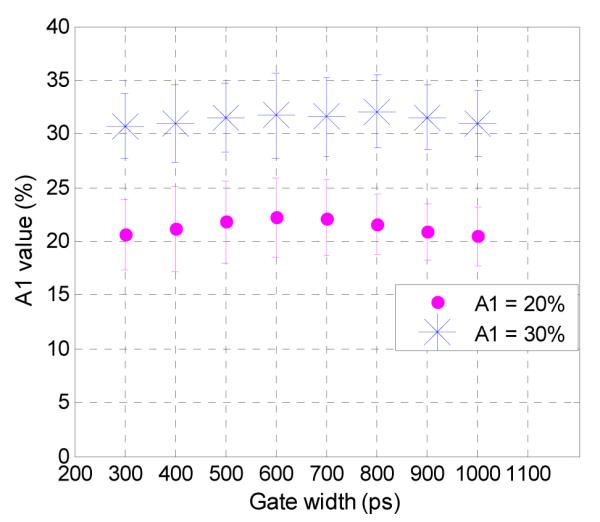
Estimation of *A*_1_ within common experimental parameters (*τ*_1_ = 300 ps, *A*_1_ = 20% and *A*_1_ = 30%, maximum photon counts = 3000).

Even though a broad range of *A*_1_ and *τ*_1_ values were tested, realistic *in vivo* experiments for intermolecular FRET are performed under the “optimal conditions” in which the maximum intensity in the region of interest is approximately 3000 photons, yielding a FRET donor fraction (*A*_1_) in the 20%–30% range and a quenched fluorescence lifetime (*τ*_1_) of 300 ps. When the fluorescence decay curves within these experimental parameters were analyzed, a consistent trend of accurate estimation of the *A*_1_ values was observed for all tested gate widths. For both tested *A*_1_ values, there is less than 2.3% absolute difference (11.4% relative) from the true value, as seen in Figure 4, with standard deviations of less than 5%. This shows that for common experimental conditions, the use of higher gate widths will still allow for accurate quantification of FRET signals.

### 3.2. Validation Using Mouse IgG in Vitro Using Varying A:D Ratios

**Figure 5:**
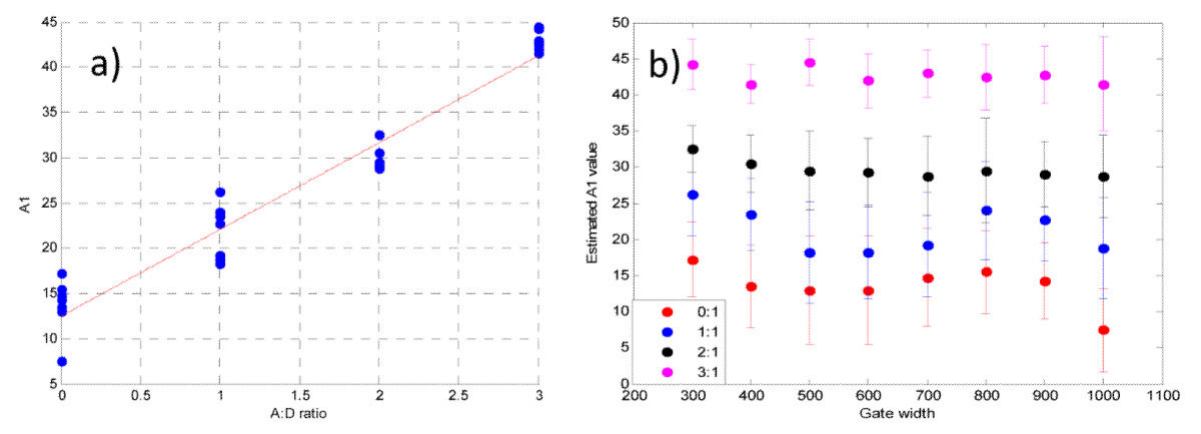
Estimated *A*_1_ values *versus* (**a**) A:D ratios and (**b**) Gate Widths.

The estimated values of *A*_1_ and *τ*_1_ for the *in vitro* case are provided in Figure 5. Note that the same data set is employed for Figure 5a,b, but a different display method is used for ease of interpretation. The overall outcome of the *in vitro* study supports the findings of the *in silico* study. Especially, the deviation from the expected values were larger for small A:D ratios in which FRET occurrence is expected to be limited. Of note, for the A:D ratio of 0:1 no acceptor is employed, and hence, no FRET is expected. However, since we use a bi-exponential model fit, a residual *A*_1_ is always obtained, typically within the range of *A*_1_ ≤ 10%. This value establishes the baseline sensitivity threshold of the approach. At A:D ratios of 1:1, the average estimated *A*_1_ is *A*_1_ = 22 ± 4% using data pooled from all gate widths. Since *A*_1_ is significantly higher than the baseline mentioned above, we can determine that *A*_1_ values for all A:D ratios above 0:1 are associated with intramolecular FRET, with increased A:D ratios leading to increased FRET occurrence (see Figure 5a). This linear trend is expected in FRET experiments, since an increase in the percentage of acceptor within the sample results in an increase in FRET interactions. In Figure 5b, the range of *A*_1_ for A:D = 0:1 was 9.75% whereas it reduced to only 3% at A:D = 3:1. Since the error is less than 10% even for the most difficult case, this means that we were still able to accurately estimate the FRET parameters for all gate widths. Note that the average value for 0:1 was 13.6%. This is a significant *A*_1_ level since no FRET should occur if there is no acceptor present. However, the conjugation of AF700 to IgG can lead to multi-exponential behavior. We have experienced similar levels of *A*_1_ at 0:1 when using the IgG pair [9], and lower levels when using the Tfn pair. Despite this, the level of FRET at 1:1 up to 3:1 is always significantly higher than the 0:1 case and exhibit the expected linear relationship *versus* A:D ratios.

### 3.3. Analysis of the Selected A:D Ratio (2:1) in Vivo

**Figure 6:**
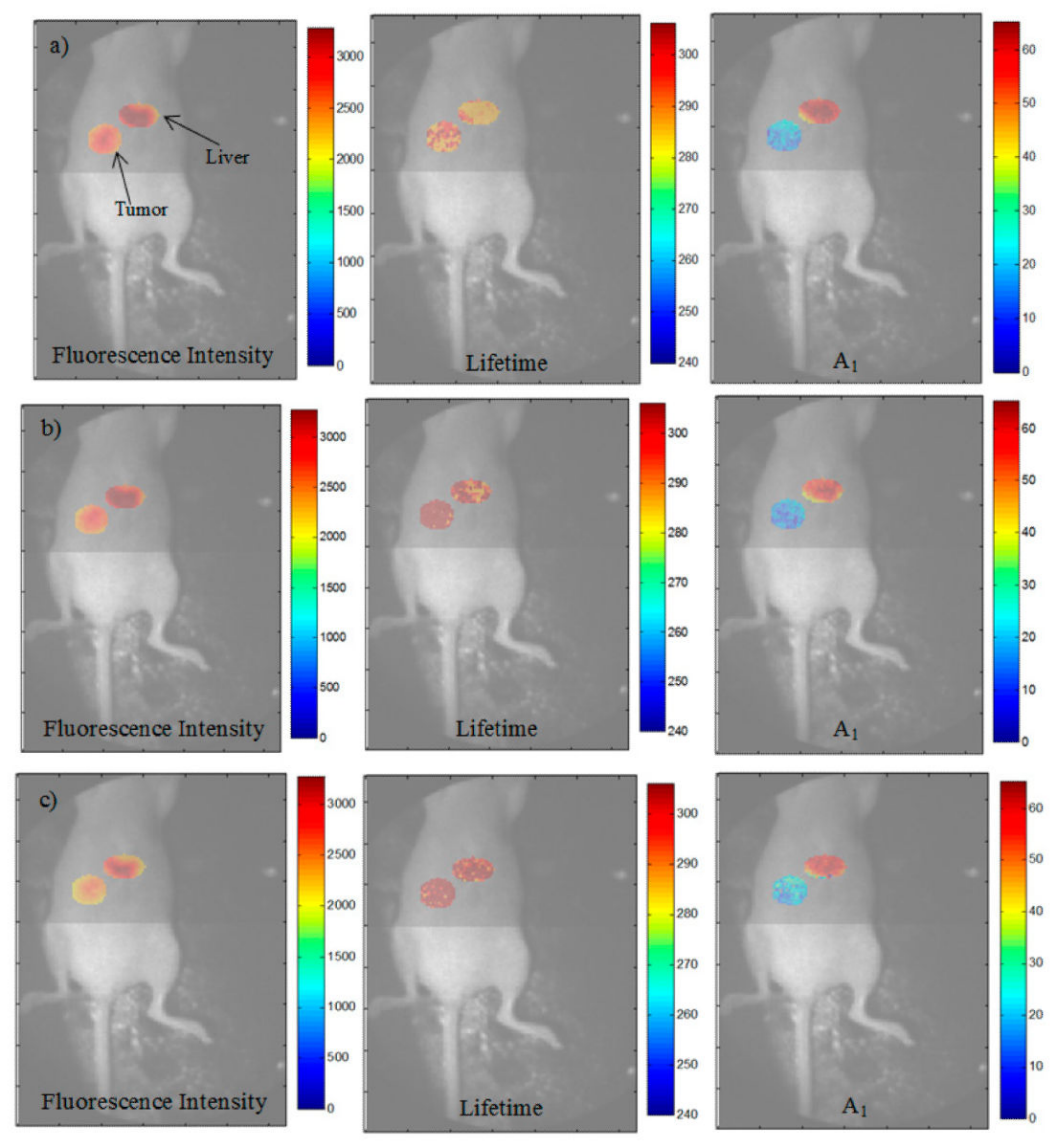
Fluorescence intensity, estimated *τ*_1_, and estimated *A*_1_ for the labeled organs of interest and gate width of (**a**) 300 ps, (**b**) 600 ps, and (**c**) 1000 ps. The photon counts were set to 3000 counts at the maximum of the TPSF during image acquisition (A:D ratio of 2:1 Tfn-AF700).

As mentioned in Section 2.5, an animal model with human T47D breast cancer xenograft was injected with transferrin probes labeled with AF700 and AF750 in a ratio of 2:1 and imaged after six hours. Visualization of FRET was performed with whole-body imaging with focus on the tumor and liver. Figure 6a,b,c represent the quantification of FRET parameters under a 300 ps, 600 ps, and 1000 ps gate width. The left, center, and middle figures show the fluorescence intensity, estimated lifetime *τ*_1_, and estimated FRET donor fraction *A*_1_ for the areas of interest. Since the system parameters were adjusted for a maximum photon count of approximately 3000 photons, the intensity within the areas is similar between the gate widths. Very little variation was seen (<50 ps) among the estimated quenched lifetimes between the two organs and across all gate widths. Overall, the range of estimated *A*_1_ values was only 5% at the liver and 6% at the tumor. As expected, the average FRET signal was high in the liver (48%–53%) and lower at the tumor (13%–19%) due to the distribution of transferrin receptors within the two tissues. The FRET signal at the tumor was slightly lower than the expected values, which may have been due to natural clearance of the labeled transferrin prior to the imaging procedures performed six hours post-injection.

**Figure 7:**
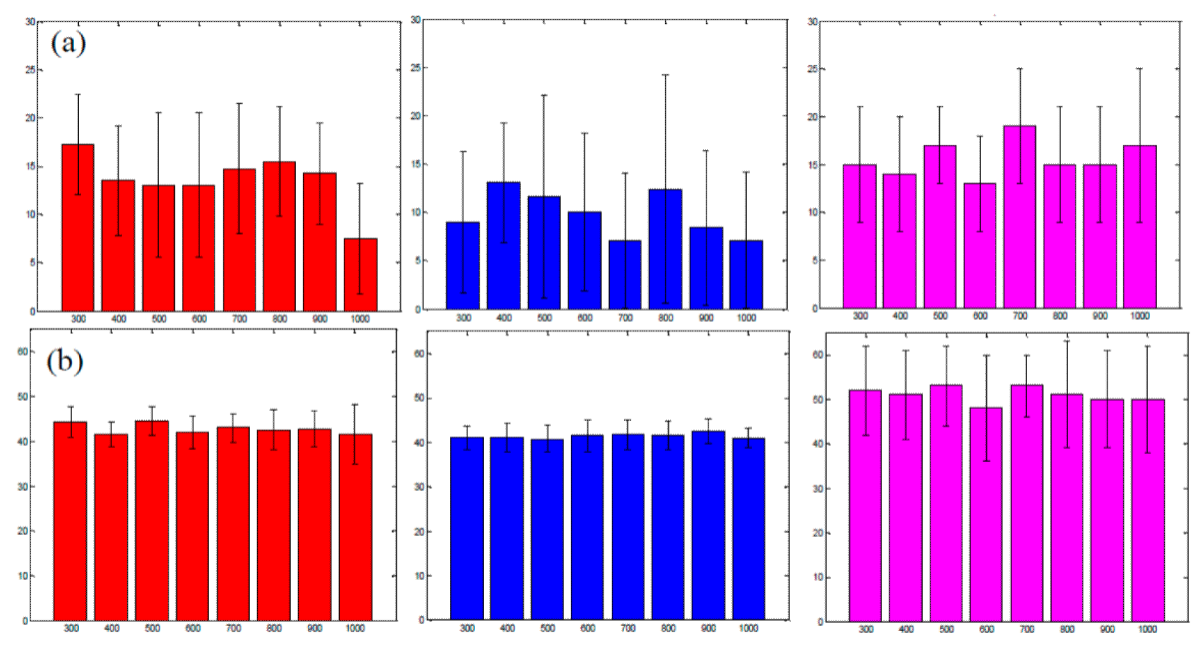
Validation of a change in gate width on (**a**) *A*_1_ = 12% and (**b**) *A*_1_ = 40% for *in vitro* (**left**), *in silico* (**middle**), and *in vivo* (**right**) cases.

In order to compare the experimental setups used herein, we chose two values of *A*_1_ (15% and 40%) that were approximately encountered in all three situations and would be indicative of FRET occurrence. Figure 7a shows the estimation of *A*_1_ for analysis of the *in vitro* well-plate experiments for A:D = 0:1, *in silico* synthetic data for a quenched lifetime of 300 ps and optimal conditions (3000 photons), and *in vivo* tumor of the mouse (right to left in the figure) for *A*_1_ = 15%. The ranges of estimation for the three data sets were 9.75%, 6%, and 6% across all gate widths. Figure 7b shows the estimations for *A*_1_ = 40% for A:D = 3:1, *in silico* data, and for the liver in the mouse. The ranges of *A*_1_ in this data set were 3%, 1.6%, and 5% respectively. The spread of the data across all gate widths is less than 10% for both analyzed values of *A*_1_, which validates the results of accurate quantification of FRET for all tested gate widths.

### 3.4. Improvement in Imaging Protocols

**Figure 8:**
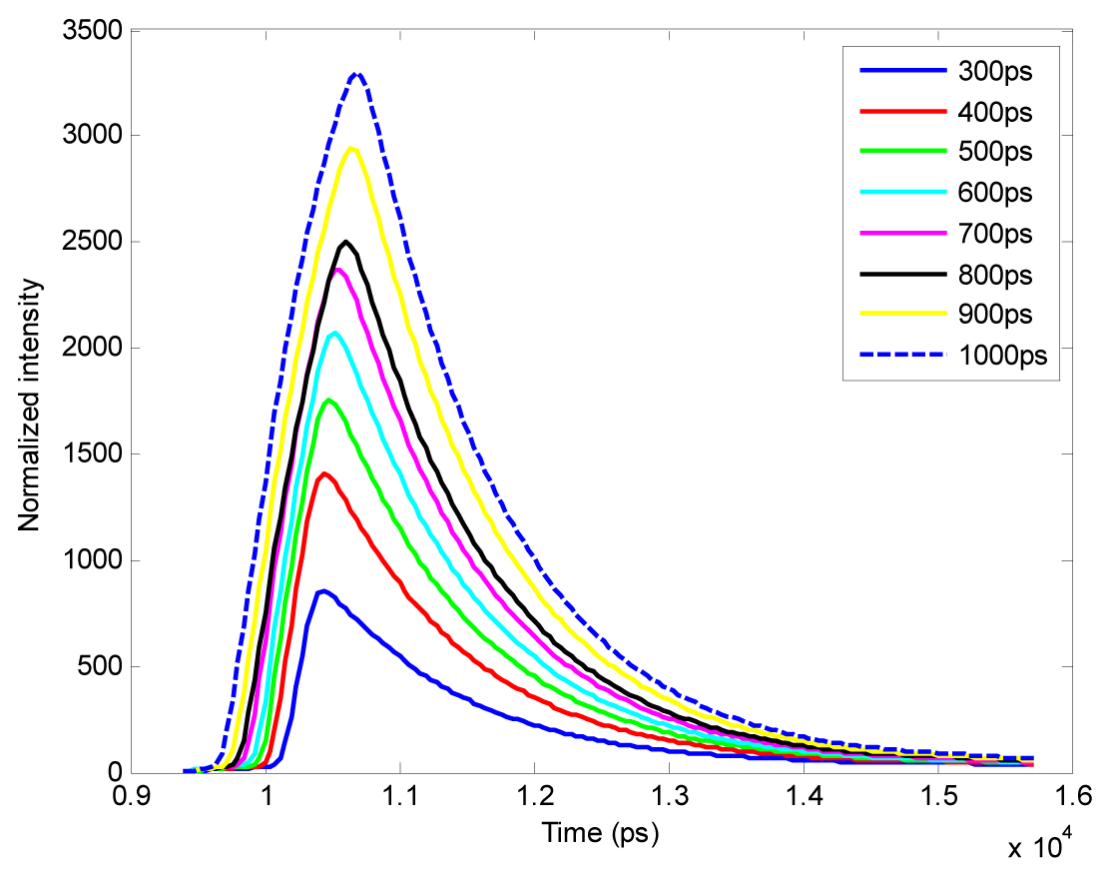
TPSFs of each gate width show increased maximum photon counts.

Upon analysis of the TPSFs created using the bi-exponential model and experimental IRFs, it was expectedly found that the total photon counts increased with the gate width, as shown in Figure 8. For instance, if we consider the low light case at 300 ps, *i.e.*, maximum peak of 1000 photons, then increasing the gate width to 1 ns without modifying any other parameters (illumination power, HV or integration time) will lead to a maximum peak of 3299 photons, which is close to optimal conditions. This is because the gate is open for a longer period of time, which allows more photons to reach the CCD during data collection. Increasing the total photon counts would be helpful in photon-starved situations in which the signal strength is weak, *i.e.*, due to low concentration or low laser power.

**Figure 9:**
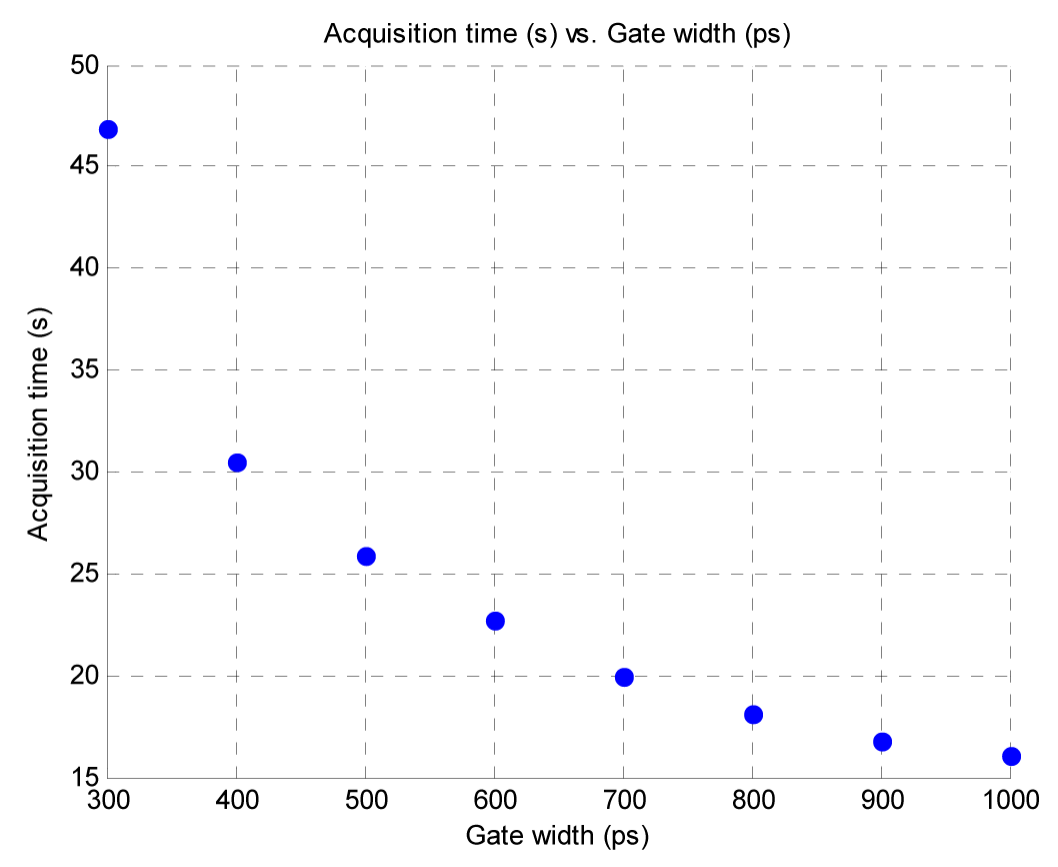
Decreased acquisition time for increased gate width.

A subsequent improvement in acquisition time was also observed, shown in Figure 9, with a three-fold decrease in acquisition time from 47 s to 16 s when the gate was increased from 300 ps to 1000 ps. This would be beneficial when large data sets need to be taken for a particular sample. Even though the image acquisition time decreases, the computation time of the bi-exponential fitting algorithms does not change because the speed is dependent upon the size of the user-defined region of interest.

Note that if decreasing the time required to complete the imaging protocols is attractive for *in vivo* imaging, the results presented herein pertain to direct wide-field imaging such as that performed with subcutaneous models. In the case of tomographic imaging [9], a model-based approach is employed that requires the computation of the forward matrix [40,41]. If increasing the gate width is not expected to significantly modify the Jacobian associated with the asymptotic part of the TPSFs, it will affect the rising portion (known as the early gates). This rising part is employed in tomography to impart resolution on the reconstructions [42–44]. As seen in Figure 8, higher gate width leads to less steep rising TPSFs, which indicates the collection of more scattered photons. Accordingly, as described by Valim *et al.* [45], it is expected that larger gate widths will increase the spatial extent of the early gate Jacobians, leading to a reduced improvement in resolution.

## 4. Conclusion

To our knowledge, this is the first work to analyze the effect of gate width on quantification of FRET in the NIR region. Lifetime-based FRET parameters were retrieved within less than 15% error in all cases investigated. The increase from 300 ps gate width to 1000 ps gate width did not lead to significant increase in errors. However, it did provide substantial increase in photon counts and reduced acquisition times. Hence, larger gate widths could improve the sensitivity of *in vivo* NIR FRET imaging, as well as reducing imaging protocol acquisition times.
